# Diverging Paths: Heterogeneity in Early Childhood Executive Function Development in Chile

**DOI:** 10.3390/brainsci16090946

**Published:** 2026-09-05

**Authors:** Camila Martínez, Pamela Soto-Ramírez, Marigen Narea

**Affiliations:** 1 Center for Advanced Studies on Educational Justice, Pontificia Universidad Católica de Chile, Santiago 7820436, Chile; psotor@uandes.cl (P.S.-R.); mnarea@uc.cl (M.N.); 2 School of Psychology, Universidad de Los Andes (Chile), Santiago 7550000, Chile; 3 School of Psychology, Pontificia Universidad Católica de Chile, Santiago 7820436, Chile

**Keywords:** executive function, early childhood, developmental trajectories, self-regulation

## Abstract

**Highlights:**

**What are the main findings?**

- Three distinct EF developmental profiles (persistently disadvantaged, normative, advantaged accelerating) emerge as early as age 3, with divergent growth rates.
- Home environment quality emerged as the strongest predictor of trajectory membership, and early cognitive development showed protective power against the lowest trajectories, whereas caregiver depressive symptoms, parenting stress, and most sociodemographic factors were not significant predictors.

**What are the implications of the main findings?**

- Enriching home learning environments before formal schooling may help reduce EF disparities.
- Given its protective role, interventions supporting early cognitive development should target infancy, not just later childhood, to prevent severe executive function delays.

**Abstract:**

Background/Objectives: Executive function (EF) is a foundational cognitive process associated with academic achievement and socioemotional adjustment. While average developmental trends are well documented, less is known about heterogeneity in EF trajectories during early childhood and the contextual factors associated with different developmental pathways. This study examined longitudinal trajectories of EF between ages 3 and 6 and their associations with family, child, and caregiver well-being, as well as the home environment. Methods: Data were drawn from the Chilean longitudinal cohort First Thousand Days (Mil Primeros Días [MPD]; *n* = 583). EF was assessed at ages 3, 5, and 6 using the Cat-Dog task. Latent profile analysis identified EF trajectories, and multinomial logistic regression examined predictors of trajectory membership. Results: Three EF trajectories were identified: Normative (54.5%), Persistently Disadvantaged (34.6%), and Advantaged Accelerating (10.8%). Home environment quality was the most consistent predictor of trajectory membership, increasing the likelihood of following the Advantaged Accelerating (RRR = 1.59) or Normative (RRR = 1.21) trajectory relative to the Persistently Disadvantaged trajectory. Early cognitive development protected against the most disadvantaged trajectory, whereas distal sociodemographic characteristics and caregiver well-being were not significant predictors of trajectory membership. Conclusions: Individual differences in EF emerge early and show divergent growth rates through age 6, with home environment quality as the strongest predictor—highlighting the value of enriching home learning environments before school entry.

## 1. Introduction

Executive function (EF) refers to a set of higher-order cognitive processes that enable individuals to regulate thoughts, emotions, and behavior in a goal-directed manner [1]. Core components of EF include inhibitory control, working memory, and cognitive flexibility, which jointly support planning, problem solving, learning, and adaptive functioning [1,2]. According to Zelazo and Müller [3], these processes are conceptualized as ‘Cool EF’, as they are primarily cognitive and decontextualized, operating with minimal emotional load. In contrast, ‘Hot EF’—such as impulse control, emotion regulation and decision making—operates in motivationally significant situations and socioemotional contexts [3]. Because all the aforementioned skills are fundamental for children’s academic achievement, socioemotional adjustment, and long-term health and well-being, EF has become a central construct in developmental science [4,5,6]. Moreover, individual differences in EF during early childhood have been shown to predict later educational attainment, fewer mental health problems, and lower levels of behavioral problems in adolescence and adulthood [5,6].

EF begins to emerge early in life and shows rapid development during the preschool years, a period characterized by substantial neurobiological maturation of the prefrontal cortex and its connectivity with parietal and subcortical regions [7,8,9]. Although EF continues to mature throughout adolescence, early childhood has been identified as a sensitive period during which individual differences in self-regulation begin to consolidate [1]. From the first year of life, infants demonstrate early forms of inhibitory control and working memory, reflected in their ability to maintain attention and inhibit previously reinforced responses [10,11]. Between ages 2 and 5, rapid improvements are observed across EF domains, including the emergence of cognitive flexibility, which enables children to switch rules and adapt behaviors to changing demands [12].

A substantial body of research has examined whether EF is best conceptualized as a unitary construct or as separable components. Evidence suggests that EF may initially function as a relatively unified cognitive capacity in early childhood, differentiating into distinct yet related components later in development [13,14]. Therefore, when examining the predictors of early cognitive development, environmental and biological factors—such as early-life stress, socioeconomic status, and prematurity—must be understood as influences on the overall architecture of self-regulation, rather than as isolated effects on individual EF components. In early childhood, before clear cognitive differentiation occurs [13,14], early adversity operates on an integrated neurobiological foundation [15]. Chronic stress or physiological vulnerability does not selectively damage working memory or inhibitory control in isolation; instead, it disrupts the broader, centralized biological systems responsible for adaptive stress responses and behavioral regulation. Consequently, environmental risks manifest as global variations in a child’s interconnected self-regulatory capacity, which in turn shape their overall developmental trajectories.

This unified perspective on early EF has critical implications for investigating developmental pathways in Latin American contexts such as Chile. While recently classified as a high-income transition economy, Chile retains deep structural socioeconomic stratification and severe income inequality. In environments with marked structural disparities, early developmental gradients are significantly steeper than those observed in high-income, egalitarian nations [16,17]. Macro-structural factors—such as highly segregated educational and health systems—shape the biological and cognitive foundations of young children long before school entry. In such a highly stratified society, examining distinct developmental trajectories allows researchers to evaluate whether the proximal home environment operates as a buffer for resilience or as a mechanism that amplifies macro-structural vulnerabilities [17]. Local longitudinal evidence underscores this dynamic; for instance, Lohndorf, Narea, and Concha [18] demonstrated in a large cohort of Chilean families that maternal characteristics and the immediate home environment in early childhood act as powerful long-term predictors of children’s executive functioning. Their findings reveal that macro-structural pressures—often manifested as elevated maternal parenting stress—directly compromise children’s global EF and working memory development. Conversely, proximal family assets, such as high parental emotional support and a robust home learning environment, serve as vital protective mechanisms that promote favorable cognitive pathways despite background sociodemographic adversity.

Although EF development is supported by important maturational processes, growing evidence suggests that it is also highly sensitive to children’s early environmental experiences and caregiving contexts [1,19,20]. Contemporary developmental frameworks emphasize that EF emerges through dynamic interactions between biological maturation and experiences with the family and home environment [1,7]. Although developmental improvements in EF are expected during early childhood, children differ substantially in both their initial levels of EF and their rates of development. Whereas some children show rapid gains in self-regulation abilities, others experience persistent difficulties that may place them at greater risk for later academic and psychosocial problems [1,20,21]. Understanding the factors that contribute to these divergent developmental pathways has therefore become a major focus of developmental research. Accordingly, a growing body of research has identified multiple family, child, and contextual factors associated with these individual differences. In particular, previous studies have highlighted the role of socioeconomic [22,23] and family conditions, child developmental characteristics, and the quality of the home environment in shaping children’s EF development and trajectories [22,23,24,25,26].

Importantly, many of the family and contextual factors associated with EF development are potentially modifiable. Evidence from systematic reviews indicates that parenting interventions promoting responsive caregiving, cognitive stimulation, and early learning opportunities can improve children’s cognitive, language, and developmental outcomes, particularly among socioeconomically disadvantaged populations [27,28]. Similarly, interventions aimed at improving caregiver well-being and reducing parenting stress may enhance the quality of parent–child interactions and indirectly support children’s developmental trajectories [29]. Together, these findings underscore the importance of identifying early predictors of EF development that may inform prevention and intervention efforts.

### 1.1. Family Characteristics

Family-level structural characteristics have been consistently associated with individual differences in children’s EF development. Socioeconomic disadvantage and lower parental education are among the most robust predictors of poorer EF across childhood, reflecting broader disparities in access to material, educational, and developmental resources [22,23,24,26]. Neuroimaging research has further shown that socioeconomic conditions, including parental education, are associated with structural variation in brain regions implicated in executive functioning, particularly the prefrontal cortex and related neural networks [26].

Caregiver psychological well-being also plays an important role. Maternal depressive symptoms during pregnancy and the postnatal period have been associated with poorer EF outcomes in offspring, potentially through reductions in caregiver responsiveness and increased family stress [30,31]. In Chile as well, Parental stress has been associated with lower EF outcomes [18]. In addition, chronic exposure to stress and adversity has been linked to alterations in the development of neural systems underlying inhibitory control, working memory, and cognitive flexibility [6,7].

Family composition may further contribute to EF development. Children living in single-parent households may be more likely to experience instability and cumulative stressors, particularly in contexts of socioeconomic vulnerability, which may adversely affect self-regulation and cognitive development [22,23]. Maternal age represents another family characteristic associated with developmental outcomes during early childhood. Younger maternal age, particularly adolescent motherhood, is frequently associated with greater socioeconomic disadvantage and lower educational attainment, factors that may shape children’s developmental opportunities and contribute to inequalities in early cognitive and EF development [24,32].

### 1.2. Child Characteristics

EF development is also shaped by child-level biological and developmental characteristics. Among these, early cognitive development has consistently been identified as one of the strongest predictors of later executive functioning, highlighting the close interplay between general cognitive abilities and emerging self-regulatory capacities [33,34,35]. Children with stronger early cognitive abilities during the early years tend to demonstrate better inhibitory control, working memory, and cognitive flexibility throughout childhood, suggesting substantial continuity between early cognitive development and later executive functioning.

Evidence regarding sex differences in executive functioning remains mixed. Although some studies have reported modest advantages for girls in specific domains such as attention and inhibitory control during early childhood, effect sizes are generally small, and findings vary across developmental stages, EF domains, and assessment methods [36,37,38]. Nevertheless, sex remains an important characteristic to consider when examining individual differences and heterogeneity in EF development.

Perinatal factors such as prematurity have also been associated with later differences in EF [39,40,41]. Children born preterm may be at greater risk for difficulties in attention, working memory, and cognitive flexibility, potentially reflecting alterations in early brain maturation and neurodevelopmental processes that support executive control [8]. These early biological vulnerabilities may persist across childhood and contribute to variability in developmental trajectories.

Moreover, child-related family composition variables, such as birth order and sibling presence, may influence opportunities for social interaction and learning within the family context. Interactions with siblings can provide natural settings for practicing turn-taking, conflict resolution, cooperation, and behavioral regulation, all of which may support the development of self-regulation and EF [42]. However, empirical evidence regarding the direct association between sibling characteristics and EF remains limited and mixed [42,43].

### 1.3. Home Environment

The home environment represents one of the most proximal contexts influencing EF development during early childhood [44]. Responsive caregiving, parental sensitivity, and cognitively stimulating interactions provide children with repeated opportunities to practice inhibitory control, working memory, and cognitive flexibility through co-regulation and guided learning experiences [1,19,24,25]. Developmental theories propose that EF emerges through everyday social interactions in which caregivers scaffold children’s ability to regulate attention, emotions, and behavior, gradually fostering independent self-regulation [45,46]. These interactions provide repeated opportunities for children to practice self-regulation in increasingly independent ways.

Parenting quality has also been associated with children’s EF through specific caregiving behaviors. Parents with stronger self-regulatory abilities are more likely to provide autonomy support, effective scaffolding, and sensitive interactions, which in turn foster children’s emerging EF skills and cognitive control [24,46].

Conversely, chaotic home environments characterized by crowding, excessive noise, instability, and inconsistent routines may hinder EF development [47]. Household chaos can reduce opportunities for sustained attention and self-regulated behavior while also diminishing parental responsiveness and emotional availability. Empirical evidence suggests that household disorganization and instability are associated with poorer inhibitory control, weaker working memory, and lower cognitive flexibility during childhood [22,23,48,49]. In contrast, stable and organized home environments may serve as protective contexts that support the development of EF during the early years.

### 1.4. The Present Study

Taken together, existing evidence suggests that EF development is shaped by a complex interplay of family, child, and home environmental factors [20]. Socioeconomic conditions, parental education, caregiver psychological well-being, early cognitive development, biological vulnerability, and the quality of the home environment have all been associated with individual differences in executive functioning during childhood [8,23,25,30,34]. Despite these advances, much of the existing literature has relied on variable-centered approaches that examine associations between predictors and average levels of EF or assess EF at a single point in time. Consequently, relatively little is known about whether children follow distinct developmental trajectories of EF and which early-life factors differentiate membership in these trajectories. This gap is particularly evident in low- and middle-income countries, where longitudinal studies examining heterogeneity in EF development remain scarce.

Identifying heterogeneous developmental pathways may provide a more nuanced understanding of how inequalities in executive functioning emerge and persist throughout early childhood. By identifying subgroups of children who follow similar developmental patterns, person-centered approaches provide a more nuanced understanding of developmental heterogeneity that traditional variable-centered analyses.

Accordingly, the present study sought to identify distinct developmental profiles of EF from preschool to early school age using a person-centered approach and to examine whether family, child, caregiver, and home environmental characteristic predicted membership in these profiles. Based on previous literature, we hypothesized that children exposed to more favorable developmental conditions—including higher parental education, stronger early cognitive abilities, and more supportive home environments—would be more likely to follow advantageous EF trajectories, whereas greater exposure to psychosocial and socioeconomic adversity would increase the likelihood of following less favorable developmental pathways.

## 2. Materials and Methods

### 2.1. Participants

The present study uses data from the Chilean longitudinal cohort study Mil Primeros Días (MPD; First Thousand Days), which was designed to characterize early caregiving environments and examine their associations with children’s developmental outcomes [50]. The MPD study began in 2019 and included five waves of data collection conducted in 2019, 2020, 2021, 2023, and 2024. Information on children and their families was collected through a sociodemographic survey administered by telephone to the child’s primary caregiver, as well as through developmental assessments conducted during home visits.

The present analyses focused on children’s EF assessed in Waves 3, 4, and 5. EF assessment using the Cat-Dog task began at Wave 3, when children were, on average, 42.25 months old, because children were too young for this assessment during the first two waves. Thus, Waves 3–5 constitute the three available EF assessment occasions in the MPD study. Children’s mean ages at each assessment were 41.25 months (SD = 1.54; range = 38–48) in Wave 3, 64.86 months (SD = 2.28; range = 59–83) in Wave 4, and 76.98 months (SD = 2.33; range = 71–82) in Wave 5.

The initial cohort included 1161 children. Because EF assessments were not available for all participants across Waves 3–5, children were classified according to the number of waves in which they had valid EF data. Among children with EF data from at least two assessment waves, 20 had no-age adjusted EF score at Wave 3 and lacked the sociodemographic information required for the subsequent predictor analyses. These participants were therefore not retained in the primary analytic sample, resulting in a final sample of 583 children. Of these, 270 had EF data for all three assessment waves and 313 had EF data for two of the three waves, which was not necessarily consecutive. The minimum two-assessment criterion was established because the primary objective was to characterize longitudinal patterns of EF performance, and a single observed assessment provides limited information regarding an individual’s developmental pattern [51,52]. The robustness of the identified profile solution to this inclusion criterion was examined in sensitivity analyses described below.

Attrition analyses compared participants included in the analytic sample (*n* = 583) with those excluded due to having EF data available for fewer than two assessment waves (*n* = 578). No significant differences were observed between groups in child age, sex, prematurity status, early cognitive development, caregiver education, caregiver depression symptoms, parenting stress. However, children included in the analytic sample had slightly older caregivers, more stimulating home environments, were less likely to live in single-parent households, and were more likely to have siblings (all *p* < 0.05).

Sociodemographic, child, and caregiver characteristics were primarily obtained from the Wave 3 household survey, corresponding to the first wave in which EF was assessed. However, some developmental covariates reflecting earlier child functioning, including cognitive development assessed using the Bayley Scales of Infant and Toddler Development (Bayley-III) [53], were obtained from Wave 1 to capture baseline developmental differences prior to EF assessment.

### 2.2. Procedure

Participants were recruited through the public primary health care system in Santiago, Chile. Municipalities in the metropolitan area of Santiago were invited to participate, and 17 of the 32 contacted agreed to take part in the study. All primary health care centers within participating municipalities were visited, and mothers attending a routine 12-month well-child visit with their infants were invited to enroll in the longitudinal study. Eligibility criteria required mothers to be at least 18 years old, Spanish-speaking, and to have a child aged 12 to 15 months at the time of recruitment (2019), with no prior diagnosis of a permanent developmental condition (e.g., auditory, visual, or motor impairments). Recruitment continued until the predetermined sample size for each municipality was achieved.

Individuals who expressed interest were subsequently contacted by telephone to confirm participation. Eligible families provided informed consent, completed a sociodemographic questionnaire, and arranged an in-home assessment. During the home visit, a trained psychologist administered a standardized battery assessing the child’s cognitive, language, social-emotional, and physical development. Each visit lasted approximately two hours, and participating families received a gift card as compensation. The same data collection procedures were implemented across the subsequent waves of the study. All study procedures were approved by the University Institutional Review Board (IRB).

### 2.3. Measures

#### 2.3.1. Executive Function

EF was assessed using the Cat-Dog task [54], an adaptation of the Hearts and Flowers task originally developed by Diamond [55]. The task was designed to assess core components of EF, including inhibitory control, working memory, and cognitive flexibility. However, evidence from the Chilean standardization study suggests that, particularly in younger children, performance is best represented by a combined inhibition–cognitive flexibility factor rather than by separate EF components [54].

The identical version of the Cat-Dog task was administered across Waves 3, 4, and 5, using the same stimuli, administration procedures, instructions, and scoring criteria at each assessment. The task consists of three phases. In the congruent phase, children are instructed to touch the screen on the same side as the cat stimulus. In the incongruent phase, children must touch the screen opposite the stimulus, which is represented by a dog. In the mixed phase, both cats and dogs are presented, requiring children to switch between rules depending on the stimulus: touching on the same side of the screen when a cat appears and on the opposite side when a dog appears.

Only the mixed phase was scored. This phase consisted of the same 33 tries at each assessment wave and places the greatest demands on EF because children must simultaneously maintain task rules, inhibit prepotent responses, and flexibly switch between response sets. The EF score corresponded to the total number of correct responds across the 33 mixed-condition trials, with higher scores indicating better EF performance. The task demonstrated excellent internal consistency in the Chilean validation study (Cronbach’s α = 0.91 [54]).

#### 2.3.2. Family Characteristics

Family characteristics were primarily obtained from the Wave 3 household survey (2023), corresponding to the first wave in which EF was assessed. Variables included caregiver educational attainment (0 = 12 years of education or less; 1= more than 12 years of education), single-parent household status (0 = no; 1 = yes), caregiver age (in years), and sibling status (0 = no siblings; 1 = at least one sibling). To examine whether the timing of caregiver and home-context assessments influenced the observed associations, sensitivity analyses were additionally conducted using the corresponding variables from Wave 1.

#### 2.3.3. Child Characteristics

Child characteristics included sex (0 = male; 1 = female), prematurity status (0 = non-premature; 1 = premature), and early cognitive development. Cognitive development was assessed in Wave 1 when children were approximately 12 months old, using the cognitive scale of the Spanish version of the Bayley Scales of Infant and Toddler Development, Third Edition [53]. The cognitive scale evaluates abilities such as attention, memory, and early problem-solving through standardized tasks administered individually by trained psychologists during the home visit. The Spanish version of the Bayley-III has demonstrated adequate reliability for the cognitive scale (α = 0.82 [50]).

#### 2.3.4. Caregiver and Home Environment

Caregiver depressive symptoms. Depressive symptoms were assessed using the Center for Epidemiologic Studies Depression Scale (CES-D [56]), a 20-item self-report measure assessing the frequency of depressive symptoms experienced during the previous week. Responses are recorded on a four-point Likert scale ranging from rarely or none of the time to most or all the time. A total raw score was computed, with higher scores indicating greater depressive symptomatology. The CES-D has been validated in Chile. Internal consistency in the present sample was high (α = 0.89 [56]).

Parenting Stress. Parental stress was assessed using the Parenting Stress Index-Short Form (PSI-SF [57]). The instrument includes 36 items that evaluate caregivers’ perceptions of stress associated with their parenting role, rated on a five-point Likert scale. Raw scores were used in the study, with higher scores reflecting greater parental stress. Internal consistency in the MPD sample was excellent (α = 0.91).

Home environment. The quality of the Home environment was assessed using the Home Observation for Measurement of the Environment Inventory (HOME-IT [58]). The HOME-IT is an observed-administered instrument completed during the home visit that evaluates the quantity and quality of cognitive, emotional, and social stimulation and support available to the child within the household. It consists of 58 items scored as 0 (not observed) or 1 (observed). Item scores are summed to obtain a total HOME score ranging from 0 to 58, with higher scores indicating a more stimulating and supportive home environment. The total HOME score was used in the analyses. Internal consistency in the MPD sample was good (α = 0.80).

### 2.4. Data Analysis

The primary objective of the study was to identify distinct developmental profiles of EF across early childhood and to examine the family, child, caregiver, and home-environment factors associated with membership in these profiles.

EF was assessed in Waves 3, 4, and 5 using the identical version of the Cat-Dog task. Because children varied slightly in age within each assessment wave and performance on the EF task is highly age-dependent during early childhood [1], raw EF scores were first adjusted for age in months separately at each wave. Specifically, raw EF scores were regressed on children’s age, and the resulting residual scores were retained as age-adjusted EF scores. To facilitate comparisons of children’s relative performance across assessment waves, residualized scores were subsequently standardized within each wave (z-scores). This procedure standardized the scale of the age-adjusted scores across assessment waves while minimizing variation attributable to age within each assessment wave. Longitudinal comparability of the underlying EF construct was evaluated separately through measurement invariance analyses, as described below.

Prior to estimating the developmental profiles, longitudinal measurement invariance of the Cat-Dog task was examined at the item level across Waves 3–5. The 33 dichotomous scored items were modeled using generalized structural equation models with a Bernoulli distribution and logit link. Configural, metric, and threshold invariance was not supported, item-level constraints were examined and selected parameters were sequentially freed to evaluate partial invariance while retaining equality constraints for the remaining items [59]. Detailed model comparisons are reported in Supplementary Table S3.

The three age-adjusted standardized EF scores were then entered as continuous indicators in the latent profile analyses. Given the study’s objective of identifying groups of children with similar patterns of EF development, Latent Profile Analysis (LPA) was employed. LPA is a person-centered approach that identifies unobserved subgroups of individuals within each profile are assumed to be relatively homogeneous, whereas differences between profiles are maximized [60]. Unlike variable-centered approaches, which focus on average associations among variables, LPA allows for the identification of heterogeneous developmental patterns within a population. This approach is particularly useful for examining developmental heterogeneity and identifying groups of children who may follow distinct pathways of EF development across early childhood [60,61].

Models specifying one through five latent profiles were estimated and compared using multiple fit indices, including the Akaike Information Criterion (AIC), the Bayesian Information Criterion (BIC), the sample-size-adjusted Bayesian Information Criterion (SABIC), entropy, and profile sizes. Lower values of AIC, BIC, and SABIC indicated better model fit, whereas entropy values above 0.80 were considered indicative of good classification accuracy [61,62]. Selection of the final solution was based on a combination of statistical fit, classification quality, parsimony, and substantive interpretability of the resulting profiles. Latent profile models were estimated using generalized structural equation modeling (GSEM) in Stata 18.

To examine whether the minimum two-assessment inclusion criterion influenced the identified profile solution, the selected LPA was re-estimated after relaxing this criterion and including all children who contributed at least one observed EF assessment (*n* = 823). GSEM estimation allowed participants with partially observed EF outcomes to contribute to the likelihood based on their available EF data. The resulting solution was compared with the primary profile solution in terms of profile-specific means and class proportions. In addition, classification certainty was examined according to the number of available EF assessments using participants’ maximum posterior probabilities of class membership.

Each participant was assigned to the latent profile corresponding to the highest posterior probability of membership [63]. Profile-specific means across assessment waves were examined to characterize the developmental patterns represented by each profile.

To examine factors associated with profile membership, multinomial logistic regression models were estimated after identification of the optimal profile solution. The profile with the lowest EF performance was selected as the reference category, and membership in the remaining profiles was compared with this reference group. Three sequential models were estimated. Model 1 included family and child characteristics: caregiver education, caregiver age, single-parent household status, sibling presence, child sex, prematurity status, and early cognitive development. Model 2 additionally included caregiver depressive symptoms (CES-D) and parenting stress (PSI). Model 3 further incorporated the quality of the home environment (HOME score) to examine whether associations observed in previous models were explained by variation in children’s home learning environments. As a sensitivity analysis, the multinomial logistic regression models were re-estimated replacing caregiver and home-context variables measured at Wave 3 with the corresponding measures collected at Wave 1. The analyses were conducted to examine whether the timing of caregiver and home-context assessments influenced the observed associations with executive function trajectories.

Results are presented as relative risk ratios (RRRs) with corresponding 95% confidence intervals. Statistical significance was evaluated using a two-tailed alpha level of 0.05. Continuous predictors were standardized (z score) before estimation to facilitate comparisons of effect sizes across variables. All analyses were conducted in Stata 18 (StataCorp LLC, College Station, TX, USA).

## 3. Results

### 3.1. Sociodemographic Characteristics of Children and Their Families

No significant differences were observed between groups in early cognitive development assessed with the Bayley Scales of Infant and Toddler Development (Bayley-III [53]), child sex, prematurity status, caregiver education, caregiver depressive symptoms, parenting stress, or home environment quality (all *p* > 0.05). The only significant difference was caregiver migrant status, with children who completed all three waves being more likely to have a migrant caregiver (χ^2^ = 5.52, *p* = 0.019). These findings suggest that attrition within the analytic sample was nonsystematic.

Table 1 presents descriptive statistics for the analytic sample. Mean EF scores increased across assessment waves, from 8.01 (SD = 5.87) in Wave 3 to 11.47 (SD = 5.49) in Wave 4 and 16.97 (SD = 6.48) in Wave 5, reflecting expected developmental gains during early childhood.

Regarding family characteristics, approximately 42% of caregivers had completed more than 12 years of education, nearly 30% of children lived in a single-parent household, and the average caregiver age was 29.82 years (SD = 6.15). Approximately two-thirds of children had at least one sibling.

With respect to child characteristics, approximately half of the sample was female (49.23%), 8.23% had been born prematurely, and the mean early cognitive development score was 40.06 (SD = 5.17). Regarding caregiver and home environment characteristics, caregivers reported a mean score of 6.03 points (SD = 6.20) of depressive symptoms and 19,53 in parenting stress (SD = 8.21). The mean HOME score was 42.87 (SD = 7.65).

### 3.2. Latent Profile Analysis

Prior to estimating the latent profile models, longitudinal measurement invariance of the Cat-Dog task was examined across Waves 3–5. Full metric invariance was not supported χ^2^ (66) = 145.69, *p* < 0.001. A partial metric invariance model was therefore estimated, allowing the loadings of six items (item 8, 15, 16, 20, 22, and 28) to vary across waves while retraining equality constraints for the remaining 27 of the 33 items loadings (81.8%). Full threshold invariance was also not supported χ^2^ (64) = 159.79, *p* < 0.001. A partial threshold model was subsequently estimated while retaining the partial metric specification. Thresholds for Items 2, 8, 9, 11, 15, 16, 20, 22, 23, and 28 were allowed to vary across waves, while equality constraints were retained for 23 of the 33 items’ thresholds (69.7%). Although the likelihood-ratio comparisons of the final partial threshold model with the partial metric model remained statistically significant, χ^2^ (44) = 63.41, *p* = 0.029, most factor loadings’ and items’ thresholds remained invariant across assessments. Overall, these findings indicated substantial, although not complete, longitudinal measurement stability of the Cat-Dog task. Detailed model comparisons are presented in the Supplementary Table S3.

Latent profile models specifying two to five profiles were estimated and compared (see Table 2). Following recommendations for latent profile analyses with moderate sample sizes, models were specified with equal variances across profiles and covariances fixed to zero to improve model stability and interpretability [60]. The optimal solution was selected based on a combination of statistical fit indices (AIC and BIC), profile size, parsimony, and substantive interpretability.

Although the AIC continued to decrease as additional profiles were extracted, the four- and five-profile solutions produced very small classes, including one profile representing fewer than 2% of the sample in the four-profile solution. Moreover, improvements in model fit were modest relative to the increase in model complexity. Consequently, the three-profile solution was retained because it provided the best balance between statistical fit, profile size, parsimony, adequate profile sizes, and substantive interpretability.

**Figure 1:**
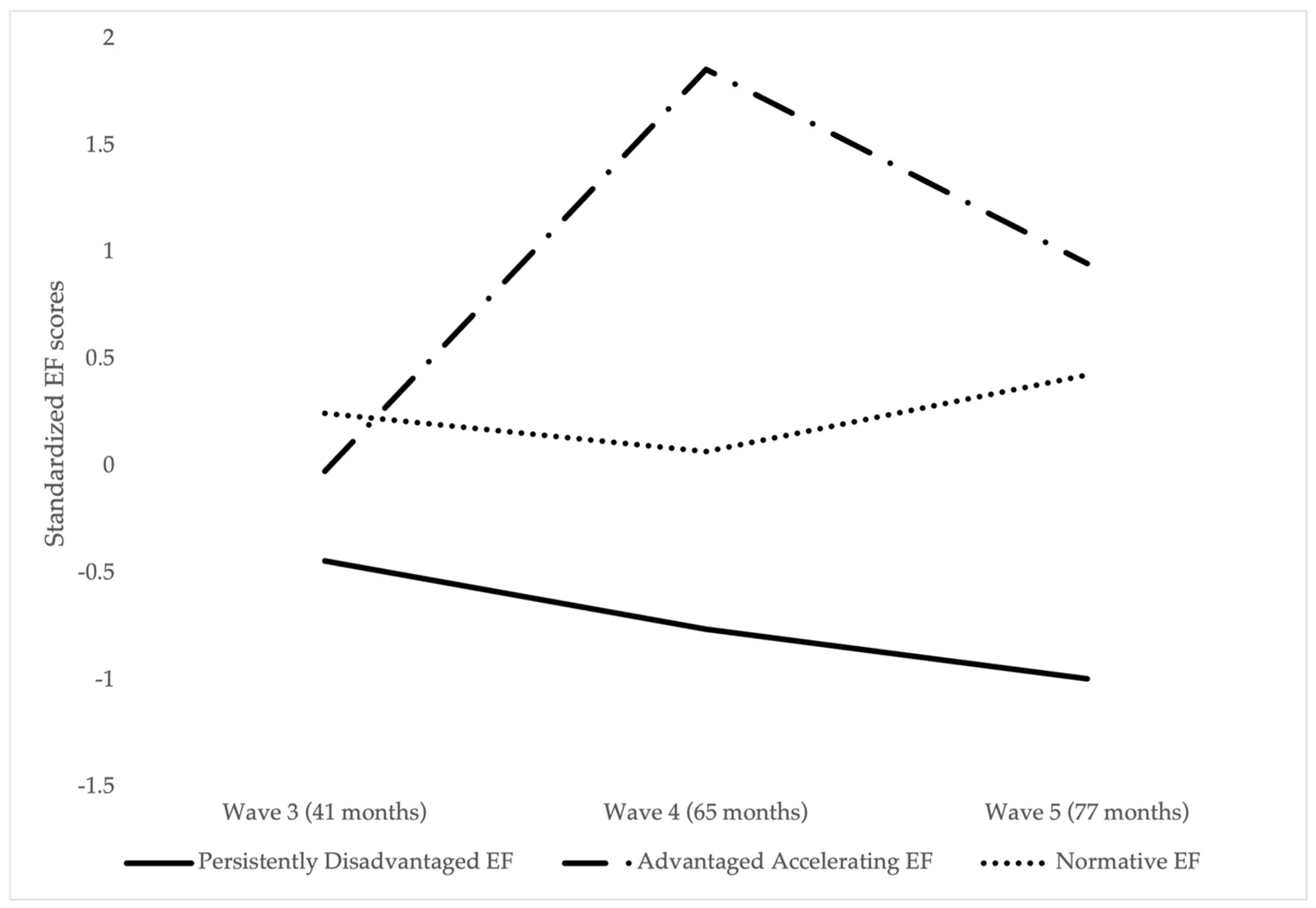
Executive function trajectories by latent profile.

The selected three-profile solution identified three distinct patterns of executive function development. The largest profile (approximately 54.5%; *n* = 318) was characterized by average EF levels across assessment waves, with steady developmental gains over time. This profile was labeled the Normative EF profile. A second profile (34.6%; *n* = 202) exhibited consistently below-average EF across all three assessment waves, indicating persistently lower EF, and was labeled the Persistently Disadvantaged EF. The smallest profile (10.8%; *n* = 63) demonstrated substantially above-average EF performance and maintained the highest scores over time. This group was labeled “Advantaged Accelerating EF”. Figure 1 displays the estimated trajectories for the three-profile solution.

Sensitivity analyses relaxing the minimum two-assessment criterion and including children with at least one observed EF assessment (*n* = 823) yielded a highly similar three-profile solution. Profile-specific means across the three assessment waves were nearly identical to those obtained in the primary analyses, although modest differences in estimated class proportions were observed. Classification certainty was lower among children contributing only one EF assessment than among those contributions two or three assessments (Supplementary Table S2).

Descriptive differences in family, child, and home environment characteristics across EF trajectory profiles are presented in Table 3. Overall, most sociodemographic characteristics were similarly distributed across profiles. No statistically significant differences were observed for child sex, prematurity status, sibling presence, caregiver education, single-parent household status, or maternal age.

Regarding developmental and family environment factors, early cognitive development showed marginal differences across profiles (F = 2.88, *p* = 0.057), with children in the Advantaged Accelerating EF tending to exhibit higher cognitive scores than children in the Persistently Disadvantaged EF. Significant differences were observed in the quality of home environment (F = 5.50, *p* = 0.004). Children in the Persistently Disadvantaged EF had lower HOME scores, whereas children in the Advantaged Accelerating and Normative EF were exposed to more favorable home learning environments.

No statistically significant differences were found across profiles in caregiver depressive symptoms (CES-D) or parenting stress (PSI), suggesting that caregiver psychological well-being was not strongly associated with EF trajectory membership in the descriptive analyses.

### 3.3. Predicting Children’s EF Trajectories from Family, Child, and Caregiver Characteristics

Multinomial logistic regression models were estimated to examine factors associated with membership in profile membership. The Persistently Disadvantaged EF served as the reference category. Results from the final model are presented in Table 4.

Among child and family characteristics, higher levels of early cognitive development were associated with an increased likelihood of belonging to the Normative EF rather than the Persistently Disadvantaged EF (RRR = 1.21, 95% CI [1.00, 1.46], *p* = 0.045). Prematurity showed a marginal association with profile membership, with children born prematurely tending to be less likely to belong to the Normative EF than to the Persistently Disadvantaged EF (RRR = 0.55, 95% CI [0.29, 1.06], *p* = 0.074).

Regarding caregiver characteristics, neither parenting stress nor depressive symptoms was significantly associated with EF trajectory membership. Similarly, child sex, maternal age, sibling presence, caregiver education, and single-parent household status were not significantly associated with trajectory membership after adjustment for other covariates.

The quality of the home environment emerged as the most consistent predictor of EF trajectories. A one-standard-deviation increase in HOME scores was associated with a 59% higher likelihood of belonging to the Advantaged Accelerating EF (RRR = 1.59, 95% CI [1.07, 2.36], *p* = 0.020) and a 21% higher likelihood of belonging to the Normative EF profile (RRR = 1.21, 95% CI [1.00, 1.45], *p* = 0.048), relative to the Persistently Disadvantaged EF.

Sensitivity analyses using Wave 1 sociodemographic variables yielded somewhat different patterns of results (Supplementary Table S1). Higher parenting stress during infancy was associated with a lower likelihood of belonging to the Normative EF trajectory (RRR = 1.61, 95% CI [0.53, 0.90], *p* = 0.006), whereas the home environment measured at 12 months was not significantly associated with trajectory membership.

## 4. Discussion

The present study explores heterogeneity in the developmental trajectories of executive function (EF) between ages 3 and 6 using longitudinal data from a Chilean cohort. Using a person-centered approach, three distinct developmental profiles were identified: an Advantaged Accelerating EF, a Normative EF, and a Persistently Disadvantaged EF. Overall, rather than indicating a rigid stabilization of individual differences, our results reveal that executive function pathways in early childhood are highly dynamic and exhibit divergent growth rates. While some sub-populations demonstrate relative stability, the emergence of the ‘Advantaged Accelerating’ profile clearly challenges the notion of a fixed cognitive destiny at age 3. This pattern aligns with Sameroff’s Transactional Model [64], which posits that development is driven by continuous, bidirectional interactions between the child’s biology and their ecological context. In this light, an accelerating trajectory may represent a developmental cascade, wherein early cognitive advantages and a highly enriched environment act synergistically to alter the developmental slope, creating a cumulative advantage that causes these children to progressively diverge from the average population growth.

Among the predictors considered, children exposed to more stimulating and supportive home environments were more likely to follow normative or advantaged EF trajectories, even after accounting for family, child, and caregiver characteristics. Early cognitive development also emerged as an important predictor of trajectory membership, suggesting that developmental differences observed during infancy may have lasting implications for executive functioning across early childhood.

The identification of three EF trajectories supports developmental perspectives emphasizing heterogeneity in early self-regulation skills. While many studies describe average age-related improvements in EF, the present findings indicate that children do not follow a single developmental pathway. Rather, meaningful differences in EF appear early and persist over time. This pattern is consistent with theoretical models proposing that early differences in cognitive control may become consolidated as children interact with their environments, potentially generating cumulative advantages or disadvantages in later development. Furthermore, the rapid growth characterized by the ‘Advantaged Accelerating’ profile warrants a nuanced interpretation that extends beyond pure cognitive capability. While the Cat-Dog task traditionally isolates ‘Cool EF’ components—such as working memory and inhibitory control in abstract settings—this accelerated trajectory may be deeply intertwined with the early maturation of affective-motivational circuits, or ‘Hot EF’ [3]. Rather than reflecting an isolated intellectual advantage, children in this profile may possess advanced socioemotional regulation. This affective maturity facilitates sustained task persistence, higher compliance, and superior frustration tolerance during structured evaluations, which fundamentally alters their observable developmental trajectories in early childhood.

One of the most consistent findings of the present study was the association between the quality of the home environment and executive function trajectory membership. Children exposed to more stimulating and supportive home environments were significantly more likely to follow Normative or Advantaged Accelerating executive function trajectories than Persistently Disadvantaged trajectories, even after accounting for family characteristics, child characteristics, and caregiver psychological well-being. Previous studies have shown similar results, with household chaos and instability being related to poorer executive functions [22,23,48,49]. This finding is consistent with developmental theories emphasizing that executive functions are shaped through repeated opportunities to engage in cognitively stimulating interactions, structured routines, and responsive caregiving. A richer home learning environment may provide children with experiences that promote attention regulation, cognitive flexibility, and inhibitory control during a period of rapid neurocognitive development.

Interestingly, once the quality of the home environment was considered, traditional sociodemographic indicators such as caregiver education, age, and family structure were no longer independently associated with membership in EF trajectory groups. Rather than suggesting that proximal characteristics of children’s daily environments are inherently more informative or important than broader structural indicators, this pattern highlights a critical mechanism of developmental mediation. When structural variables—such as parental education and socioeconomic status—lose statistical significance upon the inclusion of home-environment measures, it indicates that the effects of these distal structural determinants are likely mediated by proximal family dynamics. This cascading mechanism aligned precisely with Conger and Donnellan’s [65] (2007) Family Stress Model, which posits that macro-level socioeconomic resources operate directly by shaping caregiver well-being and the quality of stimulation available within the home. Underestimating the systemic impact of structural disparities by viewing these variables as independent or concurrent would be a conceptual error; instead, our results suggest that the proximal home environment serves as the primary behavioral and emotional conduit through which macro-structural inequalities translate into divergent executive function pathways.

Early cognitive development also emerged as an important predictor distinguishing the ‘Normative EF’ trajectory from the ‘Persistently Disadvantaged EF’ trajectory. However, it is important to note that membership in the Advantaged Accelerating profile was not predicted by early cognitive development at 12 months. These results suggest that developmental differences observed during infancy may have lasting implications for executive functioning across early childhood [34,35,66]. Specifically, early cognitive development protects against membership in the lowest trajectories. Nevertheless, it does not promote membership in the Advantaged Accelerating trajectory, for which the quality of the home environment emerged as a significant predictor. Previous studies have shown associations between early attentional processes and later executive function [34,67], suggesting that early cognitive differences may reflect underlying neurodevelopmental processes that continue to shape children’s regulatory capacities over time.

Contrary to expectations, caregiver depressive symptoms and parenting stress were not independently associated with EF trajectory membership after adjustment for family, child, and home environment characteristics. Although previous research has linked caregiver psychological well-being to children’s self-regulatory development [31,68], these associations may operate indirectly through parenting behaviors or the quality of children’s everyday learning experiences [69]. While these findings align with the hypothesis that caregiver distress operates indirectly by altering proximal parenting practices, the lack of a direct association between maternal psychological distress and EF trajectory membership must be contextualized within the specific sociopolitical and cultural reality of this Chilean sample. Because the participants were recruited from the public primary care network, they are universally covered by the national early childhood protection subsystem, Chile Crece Contigo (ChCC). This public policy framework mandates universal screening for perinatal depression and offers institutionalized, low-cost mental health support alongside targeted parenting workshops, which may actively mitigate the cascading effects of maternal distress on the household. Additionally, the cultural context of Latin American families often features extensive, informal social support networks—including extended family and community co-caregivers—that can buffer children from the direct consequences of maternal depression [70]. The presence of these institutional and communal safety nets may explain why caregiver depression and stress did not independently depress the developmental slope of executive functions. Rather than dismissing the threat of maternal distress, our results suggest that public policy frameworks like ChCC, combined with relational resilience networks, can successfully decouple caregiver psychological vulnerability from a child’s fixed neurocognitive trajectory. Nevertheless, the home environment itself did emerge as a significant and consistent predictor of EF trajectories, suggesting that proximal opportunities for cognitive stimulation may play a more direct role in shaping the development of executive function during early childhood.

Additional sensitivity analyses suggested that the timing of exposure may be relevant, as parenting stress measured during infancy (Wave 1) was associated with executive function trajectories. In contrast, the primary analyses showed that the home environment measured at age 3 was the most consistent predictor, while at Wave 1 the home environment was not significantly associated. Although these exploratory findings should be interpreted cautiously, they may indicate that different aspects of the caregiving context become salient at different developmental stages. During infancy, caregiver psychological well-being may influence the emergence of early self-regulatory capacities through sensitive and responsive caregiving. However, by the preschool years, opportunities for cognitive stimulation and structured interactions within the home environment may play a more direct role in shaping executive function development.

Several limitations should be considered when interpreting the results. First, executive function was assessed using a single task combining inhibitory control and cognitive flexibility, which may not capture the full multidimensional structure of EF. Future research could incorporate multiple indicators of executive function to better differentiate between components. In addition, although longitudinal analyses supported partial measurement invariance of the Cat-Dog task, full invariance was not achieved. Therefore, some caution is warranted when interpreting differences in EF profiles as reflecting developmental change exclusively, as some variation may reflect in item functioning across age.

Second, the analytic sample was restricted to children with executive function data from at least two assessment waves because repeated measurements were required to estimate longitudinal developmental profiles. Although attrition analyses indicated that included and excluded participants were similar across most baseline child and caregiver characteristics, small differences in caregiver age, home environment quality, and family structure were observed. Therefore, some degree of selection bias cannot be completely ruled out. However, sensitivity analyses incorporating children with at least one observed EF assessment yielded a highly similar three-profile solution, suggesting that the identified profiles were robust to the primary two assessment criteria.

Third, the current study is its exclusive focus on ‘Cool EF’ dimensions, omitting ‘Hot EF’ assessments that involve emotional arousal, reward delays, or motivational stakes. Because early childhood environment and neurodevelopment heavily impact both systems under different trajectories, the absence of ‘Hot EF’ measures restricts our ability to determine the precise role that affective regulation plays in driving these profiles. Future research should integrate mixed-method batteries containing both contextualized reward tasks and abstract cognitive measures to fully disentangle how cognitive and motivational circuits co-construct early developmental pathways. Furthermore, future studies with repeated assessments of caregiver well-being and home environment are needed to examine these developmental processes more directly.

## 5. Conclusions

Our findings highlight the importance of early developmental environments in shaping executive function trajectories during the preschool years. Three distinct patterns of executive function development were identified, suggesting that meaningful individual differences emerge early and remain relatively stable across childhood. Among the factors examined, the quality of the home environment was the most consistent predictor of trajectory membership, with children exposed to more stimulating and supportive home settings being more likely to follow normative or advantaged executive function trajectories. Early cognitive development also contributed to more favorable developmental pathways, underscoring the continuity between early cognitive skills and later executive functioning.

These findings suggest that interventions aimed at enriching home learning environments may help reduce disparities in executive function before children enter formal schooling. Strengthening opportunities for responsive interactions, cognitive stimulation, and supportive caregiving during the preschool years may therefore represent a promising strategy for fostering self-regulation and promoting healthy developmental trajectories.

## Figures and Tables

**Table 1:** Descriptive statistics of child EFs, family characteristics, child characteristics, and caregiver and home environment.

Variable	N	M/%	SD	Min	Max
**Executive function variables**					
EF Wave 3 (raw score)	382	8.01	5.87	0	32
EF Wave 4 (raw score)	539	11.47	5.49	0	31
EF Wave 5 (raw score)	515	16.97	6.48	0	33
**Family characteristics**					
Caregiver education (>12 years)	241	41.55%			
Single-parent household	173	29.67%			
Caregiver age	574	29.82	6.15	18	47
Siblings	372	64.03%			
**Child characteristics**					
Female	287	49.23%			
Premature birth	48	8.23%			
Early cognitive development	573	40.06	5.17	20	53
**Caregiver and home environment**					
CES-D (caregiver depressive symptoms)	583	6.03	6.20	0	27
PSI (parenting stress)	583	19.53	8.21	0	52
HOME total score	583	42.87	7.65	8	56

Table 1 presents descriptive statistics for the analytic sample. Mean executive function scores increased across assessment waves, from 8.01 (SD = 5.87) in Wave 3 to 11.47 (SD = 5.49) in Wave 4 and 16.97 (SD = 6.48) in Wave 5, reflecting expected developmental gains during early childhood.

**Table 2:** Fit Indices for Latent Profile Models (2–5 Profiles).

Number of Profiles	Number of Free Parameters	Log Likelihood	AIC	BIC	Smallest Profile (%)
2	10	−1993.84	4007.68	4051.37	21.9
3	14	−1981.20	3990.40	4051.55	12.9
4	18	−1972.27	3980.55	4059.18	1.9
5	22	−1961.70	3967.41	4063.51	<5

Note: AIC = Akaike Information Criterion; BIC = Bayesian Information Criterion; Lower AIC and BIC values indicate better model fit.

**Table 3:** Socio-demographic characteristics across three EF profiles.

Variables	Advantaged Accelerating EF (10.8%) *n* = 63	Normative EF (54.5%) *n* = 318	Persistently Disadvantaged EF (34.6%) *n* = 202	*p*-Value
**Family characteristics**				
Caregiver education (>12 years)	44.3%	44.3%	38.1%	0.362
Single parent household (%)	30.2%	27.7%	32.7%	0.475
Caregiver age (z) M (SD)	0.02 (0.96)	−0.01 (1.01)	0.01 (1.00)	0.968
Siblings	65.1%	64.9%	62.4%	0.832
**Child characteristics**				
Female	39.7%	49.7%	51.5%	0.255
Premature birth	9.5%	6.0%	11.4%	0.084 **•**
Early cognitive development (z) M (SD)	0.04 (1.18)	0.08 (0.94)	−0.14 (1.02)	0.057 **•**
**Caregiver and home context**				
CES-D (caregiver depressive symptoms) (z) M (SD)	0.17 (1.14)	−0.07 (0.96)	0.06 (1.02)	0.127
PSI (Parenting stress) (z) M (SD)	−0.09 (0.04)	−0.04 (0.98)	0.10 (1.02)	0.222
HOME score (z) M (SD)	0.18 (0.98)	0.08 (0.89)	−0.18 (1.14)	0.004 **

Note. M (SD) = Mean (Standard deviation). All demographic information was collected in Wave 3 (2023). Continuous variables are presented as means (standard deviations) and compared using one-way ANOVA. Categorical variables are presented as frequencies (percentages) and compared using Pearson’s chi-square tests. ** *p* < 0.01, **•** *p* < 0.10.

**Table 4:** Multinomial Logistic Regression Predicting Executive Function Trajectory Membership.

Predictors	Advantaged Accelerating EF			Normative EF		
	RRR	95% CI	*p*	RRR	95% CI	*p*
**Child and family characteristics**						
Caregiver educational attainment (>12 years)	1.18	0.63–2.22	0.610	1.24	0.84–1.83	0.285
Single parent household	0.99	0.51–1.92	0.970	0.88	0.58–1.33	0.532
Caregiver age (z)	0.96	0.69–1.33	0.808	0.99	0.81–1.20	0.887
Siblings	1.53	0.76–3.07	0.236	1.19	0.78–1.81	0.424
Female	0.60	0.33–1.11	0.100	0.92	0.63–1.33	0.645
Premature birth	0.98	0.37–2.60	0.961	0.55	0.29–1.06	0.074
Early Cognitive development (z)	1.18	0.87–1.60	0.285	1.21	1.00–1.46	0.045 *
CES-D (caregiver depressive symptoms (z)	1.23	0.89–1.69	0.211	0.90	0.73–1.11	0.327
PSI (Parenting stress) (z)	0.89	0.63–1.25	0.507	0.95	0.77–1.17	0.619
HOME score	1.59	1.07–2.36	0.020	1.21	1.00–1.45	0.048

*Note.* Results are presented as Relative Risk Ratios (RRRs) and 95% confidence intervals. Continuous predictors were standardized prior to estimation. Reference category: Persistently Disadvantaged EF. * *p* < 0.05.

## Data Availability

The data presented in this study are available on request from the corresponding author due to confidentiality agreements with the participants.

## Supplementary Materials

The following supporting information can be downloaded at: https://www.mdpi.com/article/10.3390/brainsci16090946/s1, Table S1: Sensitivity analysis using variables measured at Wave 1 (12 months) Multinomial Logistic Regression Predicting Executive Function Trajectory Membership; Table S2: Sensitivity analysis of the three-profile solutions according to the number of available Executive Function assessments; Table S3: Longitudinal measurement invariance of the Cat-Dog executive function task across Waves 3–5.

## Abbreviations

The following abbreviations are used in this manuscript:

EF	Executive function
SES	Socioeconomic status
LPA	Latent profile analysis
MPD	Mil Primeros Días (First Thousand Days study)
CES-D	Center for Epidemiologic Studies Depression Scale
PSI-SF	Parenting Stress Index–Short Form
HOME	Home Observation for Measurement of the Environment
IRB	Institutional Review Board
