# Supplementary material for: Diverging Paths: Heterogeneity in Early Childhood Executive Function Development in Chile
Source: Brain Sci. 2026 Sep 5;16(9):946. doi: 10.3390/brainsci16090946 (PMC13604083; doi:10.3390/brainsci16090946)
Supplement: Supplementary file 1 [file brainsci-16-00946-s001.zip › brainsci-4461727-supplementary.pdf]

Suplementary Table S1. Sensitivity analysis using variables measured at Wave 1 (12 months) Multinomial Logistic Regression Predicting Executive Function Trajectory Membership

Predictors	Advantaged Accelerating EF			Normative EF		
	RRR	95% CI	p	RRR	95% CI	p
**Child and family characteristics**						
Caregiver educational attainment (> 12 years)	0.84	0.39-1.81	.665	1.19	0.75-1.89	.466
Single parent household	0.87	0.36-2.10	.757	0.77	0.45-1.31	.338
Caregiver age (z)	1.12	0.76-1.66	.567	0.97	0.76-1.23	.781
Siblings	0.89	0.39-2.03	.774	0.95	0.57-1.56	.827
Female	0.49	0.24-1.03	.060	1.13	0.74-1.73	.578
Premature birth	0.69	0.19-2.56	.582	0.50	0.24-1.08	.076
Early Cognitive development (z)	1.33	0.92-1.93	.133	1.33	1.07-1.67	.012*
CES-D (caregiver depressive symptoms (z)	1.18	0.78-1.78	.445	1.17	0.91-1.51	.226
PSI (Parenting stress) (z)	0.77	0.50-1.20	.246	0.69	0.53-0.90	. 006**
HOME score	1.39	0.88-2.19	.161	0.94	0.74-1.19	.620

Note. Results are presented as Relative Risk Ratios (RRRs) and 95% confidence intervals. Continuous predictors were standardized prior to estimation. Reference category: Persistently Disadvantaged EF. Sensitivity analyses replaced single parent household, presenece of siblings, caregiver age, educational attainment, depressive symptoms, parengint stress, and home environment, measured at Wave 3 with the respective corresponding measures collected at Wave 1. Models were adjusted for the same covariates as the primary analyses.

*** p<0.001, ** p<0.01, * p<0.05, • p<0.10

Suplementary Table S2. Sensitivity analysis of the three-profile solutions according to the number of available Executive Function assessments

Panel A. Comparisons of the primery and sensitivity profile solutions

Profile	Parameter	Primary analysis: ≥ 2 EF assessmewnts (n=583)	Sensitivity analysis: ≥ 1 EF assessmewnts (n=823)
Persistently disadvantaged	Wave 3 mean	-0.32	-0.31
	Wave 4 mean	-0.60	-0.58
	Wave 5 mean	-0.76	-0.77
	Class proportion (%)	37.1%	38.0%

Advanced Accelerating	Wave 3 mean	0.06	0.06
	Wave 4 mean	1.63	1.56
	Wave 5 mean	0.85	0.88
	Class proportion (%)	12.9%	14.9%
Normative	Wave 3 mean	0.21	0.22
	Wave 4 mean	0.01	-0.06
	Wave 5 mean	0.21	0.32
	Class proportion (%)	50.0%	47.2%

Panel B. Classification certanity according to the number of observed EF assessments

Profile	n	Mean máximum posterior probability	Median	SD	≥ .70 (%)
1	220	.586	.563	.118	13.2%
2	333	.721	.709	.150	52.0%
3	270	.772	.783	.143	68.9%

Note. EF = executive function. Profile-specific means represent age-adjusted standardized EF scores. The primary analysis was base don the study's analytic sample (n = 853), whereas the sensitivity análisis included all children with at least one observed age-adjusted EF score across Waves 3-5 (n = 823). Estimated class proportions are marginal probabilites obtained from the latent profile models. Classification certainty was assessed using each participant's máximum posterior probability of class membership. Profile labels were aligned across models according to the pattern of profile-specific means rather that the numerical class labels assigned during estimation.

Suplementary Table S3. Longitudinal measurement invariance of the Cat-Dog executive function task across Waves 3-5

Model	Invariance constraints	Free parameters	Log likelihood	AIC	BIC	LR χ^2 (df)	*p*
Configural	Same factor structure across waves; loadings and thresholds freely estimated	201	−15,342.28	31,086.55	31,809.09	-	-
Full metric	All factor loadings constrained equal across waves	135	−15,415.12	31,100.24	31,585.53	145.69 (66)[a]	< .001
Partial metric	Loading for items 8, 15, 16, 20, 22, and 28 freely estimated across waves	147	−15,383.78	31,061.56	31,589.98	83.01 (54)[a]	.007
Full threshold	All thresholds constrained equal across waves, retaining partial metric constraints	83	−15,463.67	31,093.35	31,391.71	159.79 (64) [b]	<.001
Partial threshold	Thresholds for items 2, 8, 9, 11, 15, 16, 20, 22, 23, and 28 freely estimated across waves	103	−15,415.48	31,036.96	31,407.22	63.41 (44)[b]	.029

Note. AIC = Akaike Information Criterion; BIC = Bayesian Information Criterion; LR = likelihood-ratio test. All models specified a single executive-function factor at each assessment wave using the 33 dichotomously scored Cat–Dog items and a Bernoulli distribution with logit link. The partial metric model retained equality constraints for 27 of the 33 factor loadings (81.8%). The final partial threshold model retained equality constraints for 23 of the 33 item thresholds (69.7%). Lower AIC and BIC values indicate better relative fit.

[a] Compared with the configural model.

[b] Compared with the partial metric model.

Longitudinal measurement invariance of the Cat–Dog task.

Longitudinal measurement invariance was examined at the item level across Waves 3, 4, and 5 using generalized structural equation models for the 33 dichotomously scored Cat–Dog items. A one-factor model was specified at each wave. Configural invariance was examined first, followed by increasingly restrictive tests of equality of factor loadings (metric invariance) and item thresholds across waves. Because full metric invariance was not supported, item-level constraints were examined sequentially and constraints associated with the largest improvements in model fit were secuantually released. This resulted in a partial metric invariance model in which the loadings of Items 8, 15, 16, 20, 22, and 28 were allowed to vary across waves, while 27 of the 33 loadings (81.8%) remained invariant.

Full threshold invariance was subsequently tested while retaining the partial metric specification and was not supported. Sequential examination of item thresholds identified non-invariance for Items 2, 8, 9, 11, 15, 16, 20, 22, 23, and 28. These thresholds were therefore freely estimated across waves, while the remaining 23 thresholds (69.7%) were constrained to equality. The final partial threshold model showed a lower AIC than the full threshold model (31,036.96

vs. 31,093.35), whereas the BIC favored the more constrained full threshold model (31,407.22 vs. 31,391.71). Although the likelihood-ratio comparison with the partial metric model remained statistically significant, $\chi^2(44) = 63.41$, $p = .029$, the majority of factor loadings (81.8%) and item thresholds (69.7%) remained invariant across assessments. Taken together, these findings indicate substantial, although not complete, longitudinal measurement stability of the Cat–Dog task across Waves 3–5.
